# The effectiveness of the quality program Pac-IficO to improve pain management in hospitalized cancer patients: a before-after cluster phase II trial

**DOI:** 10.1186/1472-684X-13-15

**Published:** 2014-03-29

**Authors:** Carla Ida Ripamonti, Cesarina Prandi, Massimo Costantini, Elisa Perfetti, Fabio Pellegrini, Marco Visentin, Lorenza Garrino, Anna De Luca, Maria Adelaide Pessi, Carlo Peruselli

**Affiliations:** 1 Supportive Care in Cancer Unit, Department of Hematology and Pediatric Onco-Hematology, Fondazione IRCCS, Istituto Nazionale dei Tumori, Milano, Italy; 2 Palliative Care Unit Biella Hospital Italy, Biella, Italy; 3 Palliative Care Unit, IRCCS Arcispedale Santa Maria Nuova, Reggio Emilia, Italy; 4 Unit of Oncology Biella Hospital Italy, Biella, Italy; 5 Unit of Biostatistic, Fondazione Mario Negri Sud, Santa Maria Imbaro, Chieti, Italy; 6 Palliative Care Unit Vicenza Hospital, Vicenza, Italy; 7 Department of Public Health and Pediatrics Sciences, University of Turin, Turin, Italy; 8 Palliative Care Unit, Cittadella della Salute Torino, Torino, Italy

**Keywords:** Cancer, Pain, Educational program, Information of the patients, Pac-IFicO program

## Abstract

**Background:**

Cancer-related pain continues to be a major healthcare issue worldwide. Despite the availability of effective analgesic drugs, published guidelines and educational programs for Health Care Professionals (HCPs) the symptom is still under-diagnosed and its treatment is not appropriate in many patients. The objective of the study is to evaluate the efficacy of the Pac-IFicO programme in improving the quality of pain management in hospitalised cancer patients.

**Methods/design:**

This is a before-after cluster phase II study. After the before assessment, the experimental intervention – the Pac-IFicO programme – will be implemented in ten medicine, oncology and respiratory disease hospital wards. The same assessment will be repeated after the completion of the intervention. The Pac-IFicO programme is a complex intervention with multiple components. It includes focus group with ward professionals for identifying possible local obstacles to optimal pain control, informative material for the patients, an educational program performed through guides from the wards, and an organisational intervention to the ward. The primary end-point of the study is the proportion of cancer patients with severe pain. Secondary end-points include opioids administered in the wards, knowledge in pain management, and quality of pain management. We plan to recruit about 500 cancer patients. This sample size should be sufficient, after appropriate statistical adjustments for clustering, to detect an absolute decrease in the primary end-point from 20% to 9%.

**Discussion:**

This trial is aimed at exploring with an experimental approach the efficacy of a new quality improvement educational intervention.

**Trial registration:**

ClinicalTrials.gov: NCT02035098

## Background

Pain is one of the most common reasons for requesting health care service attention. The World Health Organization (WHO) estimates that 20% of individuals worldwide have some degree of chronic pain [1,2].

Despite the availability of effective analgesic drugs and of published guidelines [3-8] as well as of educational programs on the assessment and treatment of cancer-related pain [9], chronic pain is still under-diagnosed and its treatment is not adequate in many patients with both solid and haematological malignancies, in any stage of the disease [10,11].

Data from a systematic revision of the literature show that pain prevalence in cancer patients ranges from 33% in patients after curative treatment to 59% in patients on anticancer treatment, up to 64% in patients with advanced or terminal phase of disease [12].

Pain was undertreated in about 50% of the cancer patients evaluated in 26 trial by means of the Pain Management Index (PMI) [13] which evaluates the congruence between the patient’s reported level of pain and the intensity/strength of the analgesic therapy. Other surveys conducted in Italy and across Europe [10,14,15] confirmed these data, showing that pain was experienced in all cancer stages and that it was inadequately managed in a significant percentage of patients, both in early and metastatic stages, ranging between 56% and 82% and it is undertreated in oncology clinics, pain and palliative care and hospice canters [16-18].

Since 2010 pain assessment has been required by law for all patients in all Italian hospitals [19]. Major causes of undertreated pain in our country include: administration of analgesic drugs on request versus around the clock, prolonged use of no steroidal anti-inflammatory drugs [20], the employment of weak opioids, also for long periods of time, instead of strong opioids, the use of low doses of strong opioids [10,15] as well as the use of strong opioids only in metastatic and advanced disease stages, in order to avoid physical or psychological dependence, tolerance or side effects. All these factors were found to be related to malpractice among Health Care Professionals (HCPs).

The systematic management of cancer related pain is not included in the standard educational programs for clinicians in the Italian schools of medicine, whereas specific courses are available for nurses, who may also access a larger number of training courses on pain assessment and management, compared to clinicians [21-25]. On the whole, educational programs for health workers rarely meet the need for an adequate pain control in the daily practice. This may depend on different factors, such as : lack of comprehensive training programs for all the different health professionals involved in pain assessment and management, pain assessment carried out by nurses that is rarely followed by an effective evaluation and prescription by physicians, which demotivates nurses [9].

As indicated in the international guidelines, the optimal treatment of pain in cancer patients has recently been acknowledged as a major goal to guarantee the quality of care in hospitalized patients.

The availability of an effective protocol to improve the quality of pain control in hospitalized patients is mandatory in the daily practice in hospital units.

There is enough evidence supporting the potential effectiveness of the single issues (concerning the extent of information to the patients, education of HCPs and organizational support) of a program for the improvement of the quality of pain control in hospitalized patients [26-40]. The implementation and the effectiveness assessment of new pain educational programs is a mandatory issue (including education, information and organizational support) with the aim to improve pain measurement and control in all stages of oncological diseases. Such programs should have a multidisciplinary approach, thus involving nurses, physicians, psychologists, spiritual assistants and social workers, providing all health professionals with the needed information and training through a team work combining professional expertise and cure tailoring, and involving patients, who must be informed about pain and pain management and be encouraged to take an active role in their pain management [5,41].

A group of health professionals from different institutions developed a complex educational intervention aimed at improving pain control in hospitalized cancer patients. The acronym of the programme is Pac-IficO (P for Protocol, I for Information; F for Education and O for Organization). The Pac-IficO Programme includes a number of integrated interventions and is targeted to hospital wards, with the aim to significantly improve pain control in cancer patients.

The general aim of this study is assessing the effectiveness of the Pac-IFicO Programme in improving the quality of pain management in hospitalised cancer patients.

In this manuscript we report in detail the protocol used.

## Methods/design

### Objectives

#### *Primary aim*

The primary aim of this trial is to evaluate the effectiveness of the Pac-IFicO programme in improving the control of severe pain in cancer patients admitted to general medicine, oncology and respiratory disease wards of three Italian regions.

#### *Secondary aims*

The secondary aims of this study include the assessment of:

○ type and quantity of opioids administered in the wards;

○ knowledge of the ward professionals in pain management;

○ quality of pain management in cancer patients.

### Study design

This is a multicentre uncontrolled before–after cluster trial performed within hospital wards participating in the assessment of the Pac-IFicO programme.

The main objective of the Pac-IFicO programme is improving the quality of pain management in hospitalised cancer patients. The targets of the intervention are the professionals working in the hospital ward, but the outcomes of interest are assessed in clusters of patients from the hospital wards before and after the implementation of the Pac-IFicO programme.

The study is organised in different phases:

Preliminary phase

○ Identification of the eligible wards

“Before” assessment in the selected wards

○ Before evaluation according to the procedures of assessment

Implementation of the experimental intervention in the selected wards

○ Implementation of the PAC-IFicO programme

“After” assessment in the selected wards

○ After evaluation according to the procedures of assessment

### Procedures of the study

#### *Identification of the wards*

The three regional coordinators of the study obtained from local health authorities the list of all hospitals and wards of the region. For each ward the following information was collected: denomination and classification, number of beds, number of ordinary admissions per year with a primary or secondary diagnosis of tumour (ICD-IX 140-239) and their average length of stay in ward. An approximation of the punctual prevalence of the oncological patients was estimated for each ward from the last two information.

The process of identification of the eligible wards participating to this study is performed according to the following procedures:

➣ identification of all potential eligible units from the list of the hospital wards of each region (criteria 2-6 of the ward-level inclusion criteria);

➣ phone interview with the nurse coordinators on previous training on pain management received by the wards (criteria 1 of the ward-level exclusion criteria);

➣ random sampling, for each region, of the first eligible ward and subsequent evaluation of the consent from the Hospital management and the head of the ward to participate in the study. The procedure were repeated until two eligible units per region were identified.

The procedures and the dimensions of assessment for each identified ward include three levels:

Ward level

○ Information about the total consumption of opioids of the ward in the 6 months before and in the 6 months after the implementation of the Pac-IFicO programme were collected from the Hospital pharmacy.

Staff level

○ For all physicians and nurses of the ward information about qualification, years of service, training in pain management were collected.

○ Knowledge of the ward professionals in pain management were assessed before and after the implementation of the intervention by administering the Italian version of the Pain Attitudes and Knowledge Scale (PAK) [24,42];

Patient level

○ The quality of pain management is assessed before and after the implementation of the intervention by administering the APS-POK [43] to all eligible cancer patients in five weekly established sessions. The first session will be before T0) and thereafter one week apart: T7-T14-T21-T28. Each session will start at 14.00 on a midweek day. Eligible patients of each ward were identified by the nurse coordinator in the morning of the established day of assessment.

○ For all eligible cancer patient demographic and clinical information were collected. Moreover, date and time of evaluation, and causes for failed evaluation were also registered.

### End-points

#### *Primary end-point*

The primary end-point of the study is the percentage of hospitalized cancer patients with average severe pain (score 7-10) within the latest 24 hours in the after sample as compared to the before sample.

#### *Secondary end-points*

Ward level

○ Indicators about opioids administration includes: type and total amount of opioids administered in the wards to all patients;

Staff level

○ Knowledge and attitudes of the ward professionals in pain management were assessed with the Italian version of the Pain Attitudes and Knowledge Scale (PAK). The questionnaire was developed and validated by Lebovitz et al [44], and subsequently translated, adapted and validated into Italian [24,42]. The Italian version of the PAK includes 10 items, aiming to evaluate health professionals relating to their knowledge in the field of pain assessment, use of analgesics and pain management in oncological patients.

Patient level

○ The quality of pain management was assessed with the APS-POK. The questionnaire was originally developed and validated with the aim of assessing pain and pain control as perceived and rated by the patient [45]. The APS-POK is constituted of three major sections assessing: pain intensity and interference during the last 24 hours (9 items from the Pain Inventory), [46] patient’s satisfaction about pain treatment (3 items), possible prejudices preventing optimal pain management (7 items). The APS-POQ was translated and validated into Italian [43].

### Eligibility criteria

Hospital level

1. consent from the Hospital Management to participate to the study

Ward level

Including criteria

1. consent from the head of the ward to participate in the study;

2. ward classified as oncology, medicine, respiratory disease ward according to the regional classification of hospitals;

3. number of beds;

4. number of ordinary admissions with primary or secondary diagnosis of tumour (ICD-IX 140-239) >180 per year;

5. yearly average stay in hospital between 4 and 19 days;

6. punctual prevalence of patients with primary or secondary diagnosis of tumour (ICD-IX 140-239) ≥ 8 patients.

Excluding criteria

1. the ward had received or is receiving quality improvement programmes of staff education for improving pain control.

Staff level

1. affiliation to the ward;

2. informed consent to participate in the study.

Patient level

Including criteria

1. patients admitted in the ward for at least 24 hours;

2. age ≥ 18 years

3. able to fill in the questionnaire according the health care professionals judgment;

4. informed consent to participate to the study.

Excluding criteria

1 patient already assessed in one of the previous assessments.

### THE PAC-IFicO programme

In all selected wards the PAC-IFicO programme will be implemented. The **PAC-IFicO** programme shows significant differences if compared to previous Italian “Pain-free Hospital” experiences. Such differences can be listed as follows:

➣ a rigorous research in terms of methods and subsequent phases of the project;

➣ the use within hospital units of a “Program” with the same modalities of implementation;

➣ comprehensive employment of all the components of the “Program”;

➣ training based on adult education programs: education based on local barrier clearing (pre-education focus) and *peer-to-peer* training among colleagues;

➣ local organizational support interventions following the training phase;

➣ the involvement of both clinicians and nurses into the project [47];

Additional file 1: Table S1 describes the 4 phases of the **Pac-IFicO** Protocol [48,49].

Within each involved units, two Guides (a physician and a nurse) will be appointed, who will attend a four-day training course, with the aim to be enabled, in turn, to train and educate their colleagues in their unit relating to the “standard implementation” of the Program.

All the patients in the involved units will receive an information pack on pain control, during the whole protocol (three months after the completion of the training course for the health professionals).

After the training course for all the health professionals, the following organizational support interventions will be performed:

1. Monthly debate (for three months) on a **crucial clinical case** within the unit with the support of both the appointed Guides and a regional coordinator;

2. Peer to peer learning interviews among the unit staff with the Guides

3. Audit interview to the nurse coordinator of the unit three months after the completion of the training phase.

The Program includes a procedure for the evaluation of the quality of the implementation process. In particular, the following evaluations will be carried out:

➣ Evaluation of the training program; the whole process of design, implementation and learning assessment will be evaluated according to the Quality Improvement Programs. Process indicators will be used concerning the subsequent phases of both the training program for the Guides and of the standard implementation process.

➣ Evaluation of the organizational support program: all the critical clinical cases discussed within the unit will be analysed; all the support interviews among colleagues will be tracked and collected; all the audit interviews to the nurse coordinator and to the unit chief will be also analysed.

### Ethical approval

The protocol was approved by the Ethics Committee of the Promoting Center, **(CE 11/11, 18 febbraio 2011)**, and from the Ethics committees of all participating centres.

### Statistical considerations

#### *Primary end-point*

The primary outcome measurement in the present study is the percentage of hospitalized cancer patients with severe pain within the latest 24 hours (score 7-10).

### Sample size

To address the effectiveness of the Pac-Ifico Programme, a clustered sample size was estimated with an alpha error of 0.05 and a power of 80%, assuming an intraclass correlation coefficient (ICC) for the primary endpoint equal to 0.01. According to these assumptions we estimated that 10 hospital wards, with cluster size equal to 25 pre-intervention and 25 post-intervention patients corresponding to overall 500 patients, would be sufficient to detect an absolute decrease in the primary endpoint from 20% to 9%, corresponding to an odds ratio of 0.4.

### Statistical analysis

Patients’ characteristics by pre and post-intervention groups and/or by centres were reported as means, frequency and percentage, for continuous and categorical variables, respectively. Student t-test for continuous variables and Pearson chi-square for categorical variables were used to compare pre-intervention vs. post-intervention group.

To account for the nested nature of the study design (i.e. patients clustered within wards), primary and secondary endpoints were analysed with a generalized hierarchical linear model [50,51].

Results comparing pre-intervention vs. post-intervention group were expressed in terms of mean differences and odds ratios (OR) along with their 95% confidence intervals (95% CI) for hierarchical linear and logistic models, respectively. Some analyses were adjusted for known confounders as reported in details in the specific tables.

Two-sided P-values < 0.05 were considered statistically significant. All the analyses were performed using the SAS Statistical Package Release 9.1 (SAS Institute, Cary, NC, USA).

## Discussion

Although pain evaluation and management is codified both in available guidelines and, in Italy, also in the Law 38/2010, pain is still often uncontrolled both in cancer patients and in patients with non oncological chronic diseases.

Our Study aims to improve the quality of pain management in patients admitted to medicine, oncology and pneumology units in three Italian Regions: Liguria, Piemonte and Veneto.

By means of consolidated educational and organizational methodologies, the Pac-IFicO Programme actually reinforces current strategies for cancer related pain control according to international guidelines in some medicine, oncology and respiratory disease wards. Not only does the present study aim to consolidate scientific knowledge relating to clinical and non clinical recommendations contained in the object Program, but it also aims to assess the effectiveness of the whole Program as a tool to improve health care in cancer patients in pain.

The aim of this study was to assess whether the implementation of the programme may determine effective changes in the attitudes and behaviour of health professionals in view of a better practice, and whether these changes have a positive impact on the control of pain in cancer patients. The results will be analysed and interpreted at three levels: the ward level, the staff level, and the patient level, the last to evaluate the effectiveness of the induced changes in terms of an improved quality of health care.

One of the most innovative features of the present programme is the appointment of two Guides in the unit (a physician and a nurse, i.e. a small team) who will first attend a 4-day long training course, after which they will reproduce a “standard implementation” training to all their colleagues, including all the physicians and nurses within the involved unit. The hospital staff will thus become a source of continuous training, which multiplies by involving new professionals. Moreover, the monthly grand rounds on critical clinical cases, along with the peer to peer learning assessment interviews among the unit health professionals with the Guides strengthen and improve any educational initiative by including the whole involved staff.

## Competing interests

The authors declare that they have no competing interests.

## Authors’ contributions

CR, MS, CP, wrote the paper after having planned the study protocol with all the other Authors. All the Authors revised the study protocol critically. EP and FP gave their contribution in the statistical section. Moreover CP and EP were the Coordinating Research Group of the Study Protocol. CP was the Scientific Responsable of the Study Protocol. All the Authors gave final approval of the version to be published and agree to be accountable for all aspects of the work in ensuring that questions related to the accuracy or integrity of any part of the work are appropriately investigated and resolved. All the Authors read and approved the final manuscript.

## Authors’ information

*Carla Ida Ripamonti*, MD specialized in medical oncology and clinical pharmacology , Head of Supportive Care in Cancer Unit at the Fondazione IRCCS, Istituto Nazionale dei Tumori, Milano (INT), and in the past Vice Director of Pain Therapy and Palliative Care Unit of INT. Member of: European Society Medical Oncology (ESMO) Faculty Member Educational Committee; Supportive and Palliative Care; Co-Chair of the ESMO Palliative Care Working Group (PCWG); ESMO Media Ambassador; Italian Member of Multinational Association Supportive Care Cancer (MASCC); the life-time Board of Directors of the International Association Hospice Palliative Care (IAHPC).

*Maria Adelaide Pessi*, a medical oncologist mainly involved in the treatment of ovarian and gastrointestinal cancers. She actively participated in the development of national and international clinical trials, specially related with adjuvant and palliative treatment of gastrointestinal cancers and treatment of nausea and vomiting from chemotherapy. Since 2009 she is part of the supportive care team.

*Massimo Costantini*, a physician specializing in oncology, is Head of the Palliative Care Unit at the IRCCS S. Maria Nuova Hospital of Reggio Emilia (Italy). His research interests are in the areas of palliative care and quality of life. Since 2007, he is Visiting Professor in Palliative Care at the Department of Palliative Care, Rehabilitation and Policy, King’s College London (UK). He is author or co-author of more than 100 peer reviewed publications.

*Carlo Peruselli*, President of Italian Society of Palliative Care (SICP).

*Cesarina Prandi*, Research Nurse, Secretary of Italian Society of Palliative Care (SICP).

*Lorenza Garrino*, actively involved in Educational programs at the Department of Pubblic Health and Pediatrics Sciences University of Turin Italy.

*Elisa Perfetti* is a pharmacist working as Clinical Trial Coordinator at the Oncology Unit of a Hospital in the North of Italy. *Anna De Luca, Marco Visentin* are Directors of Pain Therapy and Palliative Care Units and Educational programs in the 2 comprehensive Hospitals in the North of Italy.

*Fabio Pellegrini*, is a statistician working at the Biostatistic Unit, Fondazione Mario Negri Sud. He published more than 150 papers.

## Pre-publication history

The pre-publication history for this paper can be accessed here:

http://www.biomedcentral.com/1472-684X/13/15/prepub

## Supplementary Material

Additional file 1: Table S1 Phases of the PACIFICO Program. Click here for file
