# Supplementary material for: The effectiveness of the quality program Pac-IficO to improve pain management in hospitalized cancer patients: a before-after cluster phase II trial
Source: BMC Palliat Care. 2014 Mar 29;13:15. doi: 10.1186/1472-684X-13-15 (PMC3986604; doi:10.1186/1472-684X-13-15)
Supplement: Additional file 1: Table S1 — Phases of the PACIFICO Program. [file 1472-684X-13-15-S1.doc]

**Additional file 1: Table S1. Phases of the PACIFICO Program**

The four components of the PACIFICO Program are:

1. **FOCUS GROUP with physicians and nurses of the involved units:** the aim of the focus group is to identify possible barriers within the unit preventing optimal pain control. The staff will be interviewed through the focus group.

Investigated variables within the focus group

- - “Positive” and “negative” aspects of daily health care to patients with uncontrolled pain
  - The “barriers” and “successful aspects” in the management and treatment of cancer patients
  - The feasible “strategies” to improve health care in cancer patients with uncontrolled pain

1. **informative material:** which the nurses gives to the patients at the moment of hospital admission. The aim of this material is to increase the patients’ awareness on their rights in requiring pharmacological and non pharmacological intervention and assistance in pain management and control
2. **Educational Program**: The educational program is carried out within a classroom and it involves the guides submitted to the training course (a physician and a nurse in each involved unit). As a small staff, the guides conduct a training course to all the health professionals of their unit.

Training program for the Guides

Training in the work field by the Guides

Staff training by the guides

Peer to peer training by the guides

The training program is a standard one and it is submitted to an assessment process (48); the educational material is selected according to scientific evidence-based criteria.

The training kit contains all the needed material for the staff training, along with supporting, developing, exercise materials, clinical cases to be discussed, learning assessment tests and control checklists.The Guides can conduct all the subsequent phases of the Program autonomously.

**ORGANIZATIONAL SUPPORT** (1): It includes all those **training interventions** aiming to strengthen the **organizational system:**

- - Monthly discussion (for 3 months) of a **critical clinical case** with the support of the Guides;
  - **Peer to peer** learning interviews among the unit staff with the guides

The aim of the training program is to implement the use of:

- - **Information Pack**: the hospitalized patients are supplied with an information leaflet in order to make them more and more aware of their right to require medical care for pain control and management.
  - **Pain Measurement Scales:** NRSpain assessment scale is used for all patients during all shifts, as registered in the patient’s file.
  - **Treatment Protocol Execution**

1. **Organizational Support (2)** (49): audit addressed to the staff with the presence of the nurse coordinator and the medical director of the involved hospital
